# Microarray gene expression profiling reveals antioxidant-like effects of angiotensin II inhibition in atherosclerosis

**DOI:** 10.3389/fphys.2013.00148

**Published:** 2013-06-19

**Authors:** Joshua Abd Alla, Yasser el Faramawy, Ursula Quitterer

**Affiliations:** ^1^Molecular Pharmacology Unit, Department of Chemistry and Applied Biosciences, Swiss Federal Institute of Technology ZurichZurich, Switzerland; ^2^Department of Medicine, Institute of Pharmacology and Toxicology, University of ZurichZurich, Switzerland

**Keywords:** angiotensin II, angiotensin-converting enzyme, atherosclerosis, captopril, neuroprotection, oxidative stress, perivascular nerve, vitamin E

## Abstract

Reactive oxygen species (ROS) is a significant feature of atherosclerosis but the impact of ROS on atherogenesis is not clear since antioxidants such as vitamin E have little effect on atherosclerosis development *in vivo*. To investigate the role of ROS in atherosclerosis, we used ApoE-deficient mice, and compared the treatment effect of the antioxidant vitamin E with that of the angiotensin-converting enzyme (ACE) inhibitor, captopril, because angiotensin II is a major source of ROS in the vasculature. Dihydroethidium (DHE) staining demonstrated that vitamin E and captopril both prevented the atherosclerosis-induced increase in aortic superoxide content. In contrast, seven months of vitamin E treatment retarded the development of atherosclerotic lesions by only 45.8 ± 11.5% whereas captopril reduced the aortic plaque area by 88.1 ± 7.5%. To discriminate between vitamin E-sensitive and -insensitive effects of ACE inhibition, we performed whole genome microarray gene expression profiling. Gene ontology (GO) and immunohistology analyses showed that vitamin E and captopril prevented atherosclerosis-related changes of aortic intima and media genes. However, vitamin E did not reduce the expression of probe sets detecting the aortic recruitment of pro-inflammatory immune cells while immune cell-specific genes were normalized by captopril treatment. Moreover, vitamin E did not prevent the atherosclerosis-dependent down-regulation of perivascular nerve-specific genes, which were preserved in captopril-treated aortas. Taken together, our study detected antioxidant vitamin E-like effects of angiotensin II inhibition in atherosclerosis treatment regarding preservation of aortic intima and media genes. Additional vitamin E-insensitive effects targeting atherosclerosis-enhancing aortic immune cell recruitment and perivascular nerve degeneration could account for the stronger anti-atherogenic activity of ACE inhibition compared to vitamin E.

## Introduction

Increased generation of reactive oxygen species (ROS) is a prominent feature of atherosclerosis development (Steinberg et al., 1989; Ross, 1999). The vasoactive peptide angiotensin II was identified as a major trigger of ROS generation in the cardiovascular system (Garrido and Griendling, 2009). Vice versa, inhibition of the angiotensin II system reduced the generation of oxidative stress *in vitro* and *in vivo* (Hayek et al., 1998; Wassmann et al., 2004). Concomitantly, inhibition of angiotensin II generation or angiotensin II AT1 receptor antagonism/deficiency retarded the development of atherosclerosis in animal models of atherosclerosis and patients with cardiovascular disease (Hayek et al., 1998; Yusuf et al., 2000; Abd Alla et al., 2004; Wassmann et al., 2004). From those data it was concluded that angiotensin II-dependent ROS generation contributed to the development of atherosclerosis (Keidar, 1998; Hayek et al., 2003; Wassmann et al., 2004).

On the other hand, the sole inhibition of ROS by antioxidants and/or genetic tools showed varying results in animal models of atherosclerosis (reviewed by Lönn et al., 2012). And clinical studies did not detect any reliable effect of antioxidants on the treatment or prevention of cardiovascular disease (reviewed by Schramm et al., 2012). Nevertheless, many studies confirmed that antioxidants had the potential to decrease the generation of ROS *in vitro* and *in vivo* (Ozer et al., 1993; Suarna et al., 1993; Pratico et al., 1998; Thomas et al., 2001; Gavrila et al., 2005). In view of those conflicting results between cellular and animal models, and clinical studies, the impact of ROS on the pathogenesis of atherosclerosis is still not clear.

To study the interplay between angiotensin II and ROS during the development of atherosclerosis, we applied hypercholesterolemic ApoE^−/−^ mice, which are prone to atherosclerosis and reproduce many features of atherosclerosis in patients (Piedrahita et al., 1992; Plump et al., 1992). Moreover, increased ROS generation of ApoE^−/−^ mice was confirmed by several studies (Maor et al., 1997; Hayek et al., 1998). To inhibit the generation of ROS, we used the antioxidant vitamin E, which is reported to decrease ROS and also the development of atherosclerotic plaques of ApoE^−/−^ mice when fed a normal diet (Pratico et al., 1998). The generation of angiotensin II was suppressed by the angiotensin-converting enzyme (ACE) inhibitor captopril, which has a well-established atherosclerosis-inhibitory activity in animal models and patients (Hayek et al., 1998; Abd Alla et al., 2004; McMurray et al., 2006). Treatment effects of vitamin E and captopril were compared by quantitative assessment of atherosclerotic plaques and whole genome microarray gene expression profiling. With this approach we sought to identify differences between captopril and vitamin E treatment, which could account for the weak anti-atherosclerotic effect of vitamin E *in vivo*. Our study revealed vitamin E-like effects of ACE inhibition regarding prevention of atherosclerosis-induced alterations of the aortic intima and media whereas aortic recruitment of pro-inflammatory immune cells and neurodegeneration of perivascular nerves were not sensitive to vitamin E treatment.

## Materials and methods

### Atherosclerosis treatment of ApoE^−/−^ mice

The study was performed with ApoE^−/−^ mice on a B6 (C57BL/6J) background similarly as described (Abd Alla et al., 2010). Mice were kept on a 12 h light/12 h dark regime, had free access to food and water, and were fed a standard rodent chow containing 7% fat and 0.15% cholesterol (AIN-93-based diet; without addition of tocopherol acetate). As indicated, ApoE^−/−^ mice (age 4–6 weeks) were treated for 7 months without or with captopril in drinking water (20 mg/kg; dissolved fresh every day) or tocopherol acetate (vitamin E, supplied in diet, 2000 IU/kg diet). A control group of B6 mice was also included in the study. At an age of 32–34 weeks, all mice were anesthetized with ketamine and xylazine (100/10 mg/kg), perfused intracardially with sterile PBS, aortas were isolated, rapidly dissected on ice and immediately frozen in liquid nitrogen or processed for further use. Atherosclerotic lesion area was quantified of oil red O-stained aortas by quantitative image analysis.

All animal experiments were performed in accordance with NIH guidelines, and reviewed and approved by the local committee on animal care and use (University of Zurich).

### Whole genome microarray gene expression profiling

Whole genome microarray gene expression profiling was performed essentially as described previously (Abd Alla et al., 2010). Total RNA was isolated from aortic tissue of four groups of mice: untreated ApoE^−/−^ mice, captopril-treated ApoE^−/−^ mice, vitamin E-treated ApoE^−/−^ mice and B6 mice. The RNA was processed for whole genome microarray gene expression profiling as described (Abd Alla et al., 2010). Fragmented, biotin-labeled cRNA (15 μg/gene chip) was hybridized to the gene chip (Affymetrix GeneChip MG430 2.0 Array with more than 45,000 probe sets) in 200 μl of hybridization solution in a Hybridization Oven 640 (Affymetrix) at 45°C for 16 h. GeneChips were washed and stained using the Affymetrix Fluidics Station 450 according to the instructions of the manufacturer. Microarrays were scanned with the Affymetrix GeneChip Scanner 7G, and signals were processed to a target value of 200 using GCOS (version 1.4, Affymetrix). Gene ontology (GO) analyses of microarray data were performed with GCOS/RMA processed data using GeneSpring GX software (Agilent). Data were compared between groups using the unpaired two-tailed Student's *t*-test. Probe sets with significant difference (i.e., *P* ≤ 0.01 if not otherwise stated, ≤−2-fold or ≥+2-fold difference, with call present and/or signal intensity ≥100) between treated ApoE^−/−^ mice relative to untreated ApoE^−/−^ mice were used for GO classification. Microarray data are available at the NCBI GEO database, accession numbers GSE19286 and GSE42813.

### Histology analyses and immunodetection of proteins

For immunohistology analyses, we used aortic cryo-sections prepared from vitamin E-treated, captopril-treated and untreated ApoE^−/−^ mice, and from B6 control mice. Isolated aortas of different groups were fixed with formalin (10% in PBS), dehydrated and frozen at −80°C. Frozen aortas were cut by a cryomicrotome (Microm). Aortic cryo-sections (10 μm) taken at intervals of 50 μm were prepared of the ascending aorta between the aortic sinus and aortic arch region, which is a highly susceptible region for atherosclerotic lesion development. Prior to immunohistology analysis, antigen retrieval was performed by incubation in retrieval buffer (4.2 g citric acid/2 L H_2_O, 0.05% Tween-20; pH 6.0) and heating for 30 min in a microwave. After washing with PBS, the sections were incubated in H_2_O_2_ solution (3% in PBS) for 5 min, to inactivate endogenous peroxidases. After washing steps, sections were incubated in blocking buffer (5% bovine serum albumin, 0.05% Tween-20 in PBS) for 1 h. Thereafter, sections were incubated for 1 h at room temperature with the primary antibody (dilution 1:200 in blocking buffer), followed by three washing steps with washing buffer (0.05% Tween-20 in PBS) for 5 min each to remove unbound antibody. After incubation with the secondary antibody-peroxidase-conjugate against rabbit [F(ab)_2_-fragments, dilution 1:500 in blocking buffer] followed by washing steps, the detection of bound antibody was performed by an enzyme-substrate reaction using DAB (3,3,'diaminobenzidine tetrahydrochloride) as substrate (DAB Enhanced Liquid Substrate System; Sigma). The following antibodies were used: anti-CCR9 (raised in rabbit against recombinant CCR9); anti-Cd8b (raised in rabbit against a recombinant protein corresponding to amino acids 22–175 of Cd8b); anti-neuropeptide Y (raised in rabbit against recombinant neuropeptide Y); anti-Pln (raised in rabbit against a peptide corresponding to amino acids 2–25 of Pln); anti-SNAP25 (raised in rabbit against recombinant SNAP25); anti-Sprr3 (raised in rabbit against recombinant Sprr3). Aortic superoxide content was determined by dihydroethidium (DHE) staining of aortic cryosections followed by quantitative assessment of superoxide-generated fluorescence similarly as described (Edwards et al., 2013). Quantification of immunohistology data was done on four animals/group, using ten sections (10 μm) per mouse taken at intervals of 50 μm of the ascending aorta between the aortic sinus and aortic arch region, similarly as described (Abd Alla et al., 2004). Quantitative assessment of aortic proteins by immunoblotting was performed essentially as described (Fu et al., 2013).

For atherosclerotic lesion quantification, isolated aortas were opened longitudinally, fixed in formalin (10% in PBS, 21 h) and stained with oil red O. For oil red O staining, a stock solution of oil red O (0.3 g oil red O in 10 ml of 2-propanol) was prepared and filtered (Whatman grade 1 filter paper). The stock solution was freshly diluted 6:4 with H_2_O followed by sterile filtration (0.2 μm). For oil red O staining, formalin-fixed aortas were rinsed with H_2_O and incubated with 2-propanol (70%). The internal lumen of the pinned aortas was stained with the diluted oil red O solution for 20 min, followed by a brief incubation in 2-propanol (70%) and rinsing with H_2_O. Oil red O-stained atherosclerotic lesion area was quantified by image analysis with SigmaScan Pro software.

Unpaired, two-tailed Student's *t*-test was used to calculate *P*-values. Analysis of variance was performed with Prism (GraphPad). Statistical significance was set at a *P*-value of < 0.05, unless indicated otherwise.

## Results

### Angiotensin-converting enzyme inhibition by captopril or antioxidant treatment with vitamin E retarded the formation of atherosclerotic lesions in ApoE^−/−^ mice

**Figure 1:**
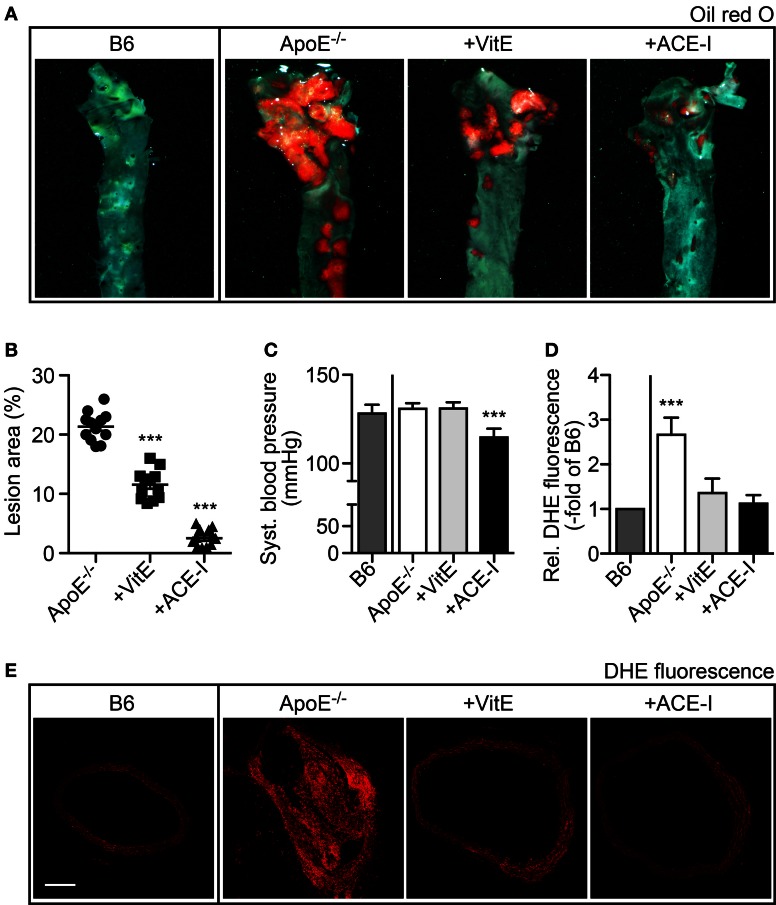
**Angiotensin-converting enzyme inhibition by captopril and antioxidant treatment with vitamin E retarded the formation of atherosclerotic plaques in ApoE^−/−^ mice. (A)** Representative oil red O-stained aortas isolated from a 34 week-old untreated B6 control mouse, a 34 week-old untreated ApoE^−/−^ mouse, a vitamin E-treated ApoE^−/−^ mouse (+VitE), and an ACE-inhibitor-treated ApoE^−/−^ mouse (+ACE-I). **(B)** Atherosclerotic lesion area was quantified by quantitative image analysis of oil red O-stained aortas isolated from 32–34 week-old ApoE^−/−^ mice, vitamin E-treated ApoE^−/−^ mice and ACE-inhibitor-treated ApoE^−/−^ mice (*n* = 12 mice/group; ^***^P < 0.0001 vs. ApoE^−/−^). **(C)** Systolic blood pressure of different treatment groups of mice (±s.d.; *n* = 5 mice/group; ^***^P < 0.0001 vs. B6). **(D)** Detection of aortic ROS *in situ* by dihydroethidium (DHE) staining of aortic sections and quantitative assessment of relative fluorescence levels generated by reaction of dihydroethidium with superoxide (±s.d.; *n* = 5 mice/group; ^***^P = 0.0006 vs. B6). **(E)** Representative DHE-stained aortic sections from a 34 week-old untreated B6 control mouse, a 34 week-old untreated ApoE^−/−^ mouse, a vitamin E-treated ApoE^−/−^ mouse (+VitE), and an ACE-inhibitor-treated ApoE^−/−^ mouse (+ACE-I); bar 200 μm.

To investigate the antioxidant effect of angiotensin II inhibition on atherosclerosis, we compared the treatment effect of the ACE inhibitor captopril with that of the antioxidant vitamin E. As disease model of atherosclerosis, we used hypercholesterolemic ApoE^−/−^ mice. After treatment for 7 months, aortas were dissected and atherosclerotic lesion area of oil red O-stained aortas was quantified of vitamin E-treated and captopril-treated ApoE^−/−^ mice relative to untreated ApoE^−/−^ mice (Figure 1A). Aortic lesion quantification showed that vitamin E had retarded the development of atherosclerotic plaques leading to a decrease in atherosclerotic lesion area by 45.8 ± 11.5% (Figures 1A,B). In agreement with previous data (Abd Alla et al., 2010), angiotensin II inhibition by captopril had largely prevented the development of atherosclerotic plaques of ApoE^−/−^ mice (Figure 1A), i.e., the atherosclerotic plaque area was reduced by 88.1 ± 7.5% in captopril-treated mice compared to untreated ApoE^−/−^ mice (Figure 1B). As a control, captopril had significantly reduced the systolic blood pressure of ApoE^−/−^ mice from 130.8 ± 3.2 mmHg to 114.6 ± 5.0 mmHg whereas vitamin E had no effect on blood pressure (Figure 1C).

### ACE inhibition by captopril and vitamin E treatment prevented the increase in aortic superoxide content of ApoE^−/−^ mice

We asked whether the aortic ROS production was affected by captopril or vitamin E treatment. The aortic superoxide content was determined *in situ* by DHE staining, which reacts with superoxide to form a fluorescent product, 2-hydroxyethidium (Zhao et al., 2003; Edwards et al., 2013). Quantitative fluorescence evaluation of DHE-stained aortic sections revealed a significantly increased superoxide content of untreated ApoE^−/−^ aortas, i.e., superoxide-dependent fluorescence was increased 2.7 ± 0.4-fold compared to B6 control mice (Figures 1D,E). In contrast, vitamin E and captopril largely prevented the increase in aortic ROS of ApoE^−/−^ mice, because the fluorescence of DHE-stained aortas from vitamin E- and captopril-treated ApoE^−/−^ mice was not significantly different from B6 control level (Figures 1D,E). Thus, vitamin E and captopril both exerted antioxidant-like effects *in vivo*, in atherosclerosis-prone ApoE^−/−^ mice.

### Whole genome microarray gene expression profiling of atherosclerosis treatment with vitamin E and captopril revealed concordantly regulated aortic genes

**Figure 2:**
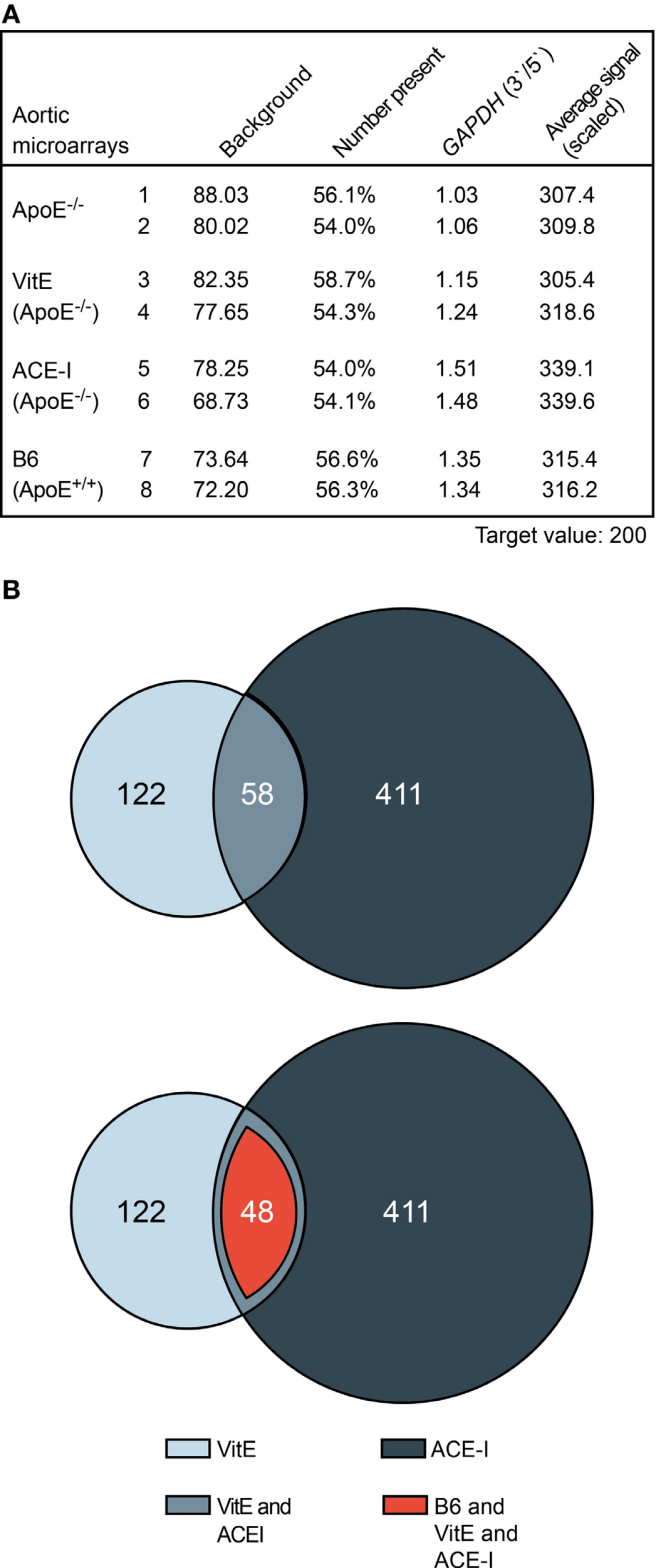
**Whole genome microarray gene expression profiling of atherosclerosis treatment with vitamin E and captopril revealed concordantly regulated aortic genes. (A)** Characteristics of aortic microarrays used for data analysis. Two different gene chips are presented of each study group. For each gene chip (Affymetrix GeneChip MG430 2.0 Array with more than 45,000 probe sets), RNAs from three different aortas were pooled. **(B)** The upper panel presents a Venn diagram illustrating that 58 significantly different probe sets showed concordant regulation between aortas isolated from vitamin E-treated (VitE) and captopril-treated (ACE-I) ApoE^−/−^ mice relative to untreated ApoE^−/−^ mice. Probe sets with significant difference (*P* ≤ 0.01; ≤-2-fold or ≥2-fold difference, signal intensity ≥100 and/or call present) between vitamin E-treated and non-treated ApoE^−/−^ mice (180), and captopril-treated and non-treated ApoE^−/−^ mice (469) were identified and used for further analysis. The lower panel illustrates that 82.8% (i.e., 48 probe sets) of concordantly regulated probe sets between vitamin E- and captopril-treated ApoE^−/−^ aortas showed normalization toward B6 control level.

To investigate gene expression changes induced by the treatment of ApoE^−/−^ mice with vitamin E and captopril, respectively, we performed whole genome microarray gene expression profiling of aortic tissue isolated from vitamin E-treated and captopril-treated ApoE^−/−^ mice relative to untreated ApoE^−/−^ mice with prominent atherosclerotic plaques. As a control, aortic tissue of healthy, non-transgenic B6 mice was also analyzed. Gene expression data showed a uniform quality of the hybridized microarray gene chips from four different groups of mice as evidenced by a comparable number of probe sets present (Figure 2A). RNA integrity of all groups was demonstrated by the comparable 3′/5′ signal intensity ratios of probe sets detecting housekeeping genes such as *GAPDH* (Figure 2A).

Data filtering was performed to detect significantly different probe sets between treated and non-treated ApoE^−/−^ mice. Data filtering showed that vitamin E treatment had significantly altered the signal intensity of 180 probe sets whereas ACE inhibitor treatment with captopril had significantly altered 469 probe sets (Figure 2B). Among significantly different probe sets, more than 30% of vitamin E-regulated probe sets, i.e., 58, showed concordant regulation with probe sets affected by ACE inhibitor treatment (Figure 2B, upper panel). On the other hand, 14% of captopril-regulated probe sets showed concordant regulation with probe sets regulated by vitamin E treatment (Figure 2B, upper panel). Together these results indicate that a significant proportion of ACE inhibitor-regulated probe sets are sensitive to treatment with the antioxidant vitamin E.

**Figure 3:**
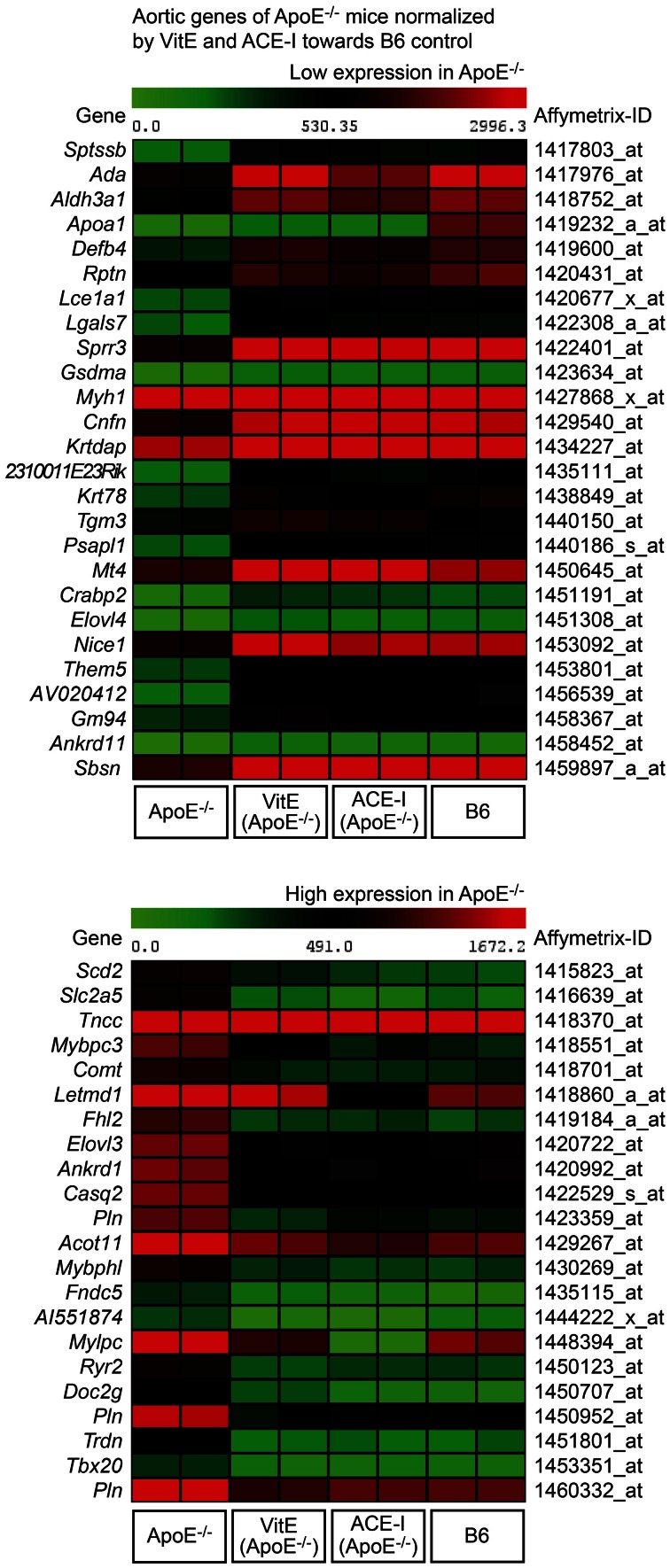
**Identification of atherosclerosis-related aortic genes of untreated ApoE^−/−^ mice, which were normalized by vitamin E (VitE) and ACE inhibitor (ACE-I) treatment toward B6 control level.** The first panel presents a heat map of genes with low expression in untreated ApoE^−/−^ aortas, and the second panel presents probe sets with high expression in untreated ApoE^−/−^ mice (*P* ≤ 0.01, and ≥2-fold, or ≤-2-fold difference).

Concordantly regulated genes between vitamin E-treated and captopril-treated ApoE^−/−^ mice could be of significant relevance for the pathogenesis of atherosclerosis because more than 82% (i.e., 48) of those commonly regulated probe sets were normalized toward B6 control level (Figure 2B, lower panel). Among concordantly regulated probe sets, which were normalized toward B6 control level, 26 probe sets had significantly lower signal intensities in untreated ApoE^−/−^ aortas (Figure 3, upper panel) whereas 22 probe sets showed a higher expression in untreated ApoE^−/−^ mice (Figure 3, lower panel). We focused on those 48 probe sets, which were normalized toward B6 control level, to gain insight into mechanisms underlying the antioxidant-sensitive component of atherosclerosis treatment by the ACE inhibitor captopril.

### Vitamin E and ACE inhibitor treatment maintained the integrity of aortic intima genes of atherosclerosis-prone ApoE^−/−^ mice

**Figure 4:**
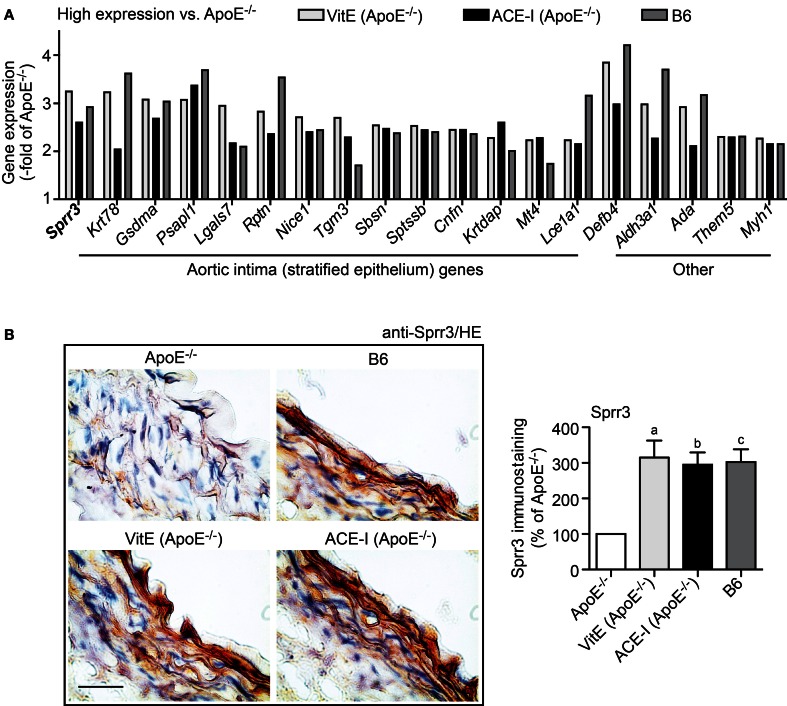
**Vitamin E and ACE-inhibitor treatment maintained the integrity of aortic intima genes of atherosclerosis-prone ApoE^−/−^ mice. (A)** Microarray gene expression data of concordantly regulated genes with high expression in vitamin E-treated (VitE) and ACE-inhibitor treated (ACE-I) ApoE^−/−^ aortas relative to untreated ApoE^−/−^ mice (≥2-fold higher signal intensity; *P* ≤ 0.01) and normalization toward B6 control level. GO analysis classified the majority of those genes with high expression as stratified epithelial genes of the aortic intima, which could preserve the biomechanical barrier function of the aortic intima. Relative gene expression of concordantly regulated genes is presented as—fold of untreated ApoE^−/−^ mice. **(B)** Left panels: immunohistological detection of Sprr3 with anti-Sprr3 antibodies validated microarray data and showed down-regulation of Sprr3 in the aorta of a 34 week-old untreated ApoE^−/−^ mouse relative to an age-matched B6 mouse (upper panels). The Sprr3 protein was preserved by vitamin E treatment and ACE-inhibitor treatment (lower panels). Nuclei were counterstained with hematoxylin, HE (bar: 40 μm). The right panel shows quantitative evaluation of immunohistology data from four mice each (±s.d.; *n* = 4; ^a^*P* = 0.0029; ^b^*P* = 0.0014; ^c^*P* = 0.0015 vs. ApoE^−/−^).

GO analysis was performed of concordantly up-regulated probe sets from vitamin E- and captopril-treated ApoE^−/−^ aortas. GO analysis identified that the majority of genes with ≥2-fold higher expression compared to untreated ApoE^−/−^ aortas were associated with stratified epithelium (Figure 4A). Moreover, expression of those genes was normalized toward B6 control level (Figure 4A). According to a previous study, genes associated with stratified epithelium are characteristic of the aortic intima and could protect the aortic intima against biomechanical stress (Young et al., 2005).

Immunohistology analysis confirmed the microarray data for small proline-rich protein 3 (*Sprr3*) as a typical gene, which was more than 3-fold up-regulated by vitamin E (Figure 4B). Sprr3 showed prominent localization in the aortic intima and adjacent media of a vitamin E-treated and ACE inhibitor-treated ApoE^−/−^ mouse, respectively, whereas the Sprr3 protein was barely detectable in the aorta of an untreated ApoE^−/−^ mouse (Figure 4B). Immunohistology also indicated that aortic Sprr3 was maintained at B6 control level by vitamin E and captopril treatment (Figure 4B). Together these findings are compatible with the notion that vitamin E treatment and ACE inhibitor treatment protected the aortic intima of atherosclerosis-prone ApoE^−/−^ mice against ROS-mediated damage.

### Antioxidant and ACE inhibitor treatment preserved the contractile phenotype of aortic media

**Figure 5:**
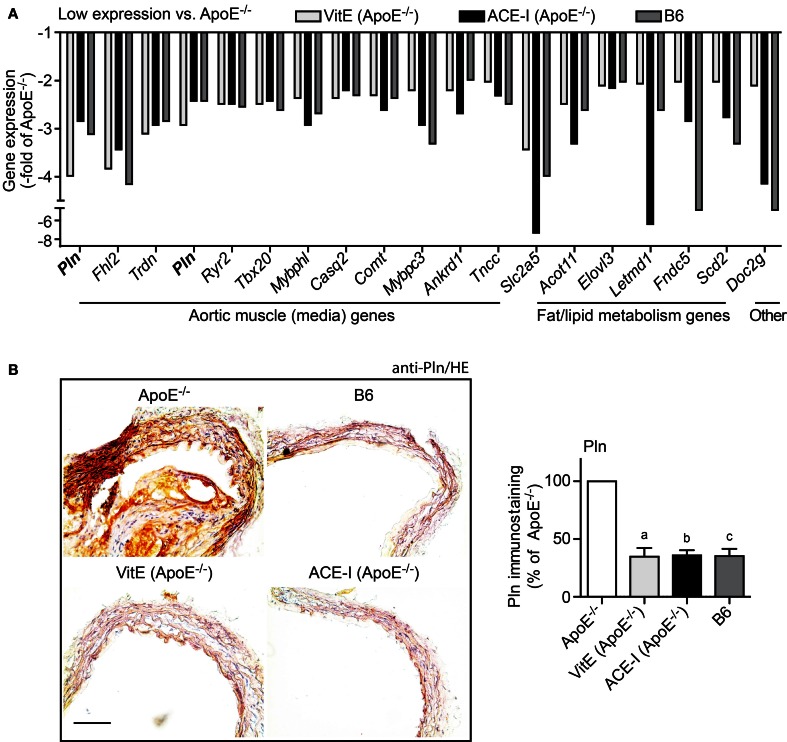
**Vitamin E and ACE inhibitor treatment preserved the contractile phenotype of the aortic media. (A)** Microarray gene expression data of concordantly regulated genes with low expression in vitamin E-treated (VitE) and ACE inhibitor-treated (ACE-I) ApoE^−/−^ aortas (≤-2-fold lower signal intensity than that of untreated ApoE^−/−^ aortas; *P* ≤ 0.01) and normalization toward B6 control level. GO analysis classified the majority of those genes with low expression as aortic muscle genes of the aortic media, which indicates that vitamin E and ACE-inhibitor treatment could prevent the atherosclerosis-related transition of the contractile phenotype of aortic smooth muscle cells to the synthetic phenotype. Relative gene expression of concordantly regulated genes is presented as—fold of untreated ApoE^−/−^ mice. **(B)** Left panels: immunohistological detection of phospholamban (Pln) with anti-Pln antibodies validated microarray data and showed up-regulation of Pln in the ascending aorta of a 34 week-old untreated ApoE^−/−^ mouse relative to an age-matched B6 mouse, a vitamin E-treated ApoE^−/−^ mouse and an ACE-inhibitor-treated ApoE^−/−^ mouse (bar: 100 μm; nuclei were counterstained with hematoxylin, HE). The right panel shows quantitative evaluation of immunohistology data from four mice each (±s.d.; *n* = 4; ^a^*P* = 0.0004; ^b^*P* = 0.0001; ^c^*P* = 0.0003 vs. ApoE^−/−^).

We also performed GO analysis of concordantly regulated probe sets with low expression in treated ApoE^−/−^ mice compared to untreated ApoE^−/−^ mice. GO analysis identified aortic muscle-specific genes as the major category of probe sets (12 probe sets), which showed a significantly lower expression in treated ApoE^−/−^ mice relative to untreated ApoE^−/−^ mice (Figure 5A). Notably, vitamin E and captopril preserved most aortic media-specific genes at B6 control level (Figure 5A).

Microarray data were confirmed by immunohistology analysis, which demonstrated the significant up-regulation of the muscle-specific phospholamban (Pln) in the ascending aorta of untreated ApoE^−/−^ mice (Figure 5B). In contrast, phospholamban staining was near B6 control level in vitamin E-treated and captopril-treated aortas (Figure 5B). Moreover, immunohistology analysis of phospholamban detected the proliferation of phospholamban-positive smooth muscle cells in the aortic media of the atherosclerotic aorta (Figure 5B). This finding is significant because proliferation of vascular smooth muscle cells is a characteristic feature of atherogenesis marking the transition of the contractile phenotype of aortic smooth muscle cells to the synthetic phenotype (Denger et al., 1999). Thus, antioxidant-like effects of the ACE inhibitor captopril could prevent the switch from the contractile to the synthetic phenotype of smooth muscle cells within the aortic media.

### Pro-inflammatory immune cell recruitment into the atherosclerosis-prone aorta was sensitive to ACE inhibition but insensitive to vitamin E treatment

**Figure 6:**
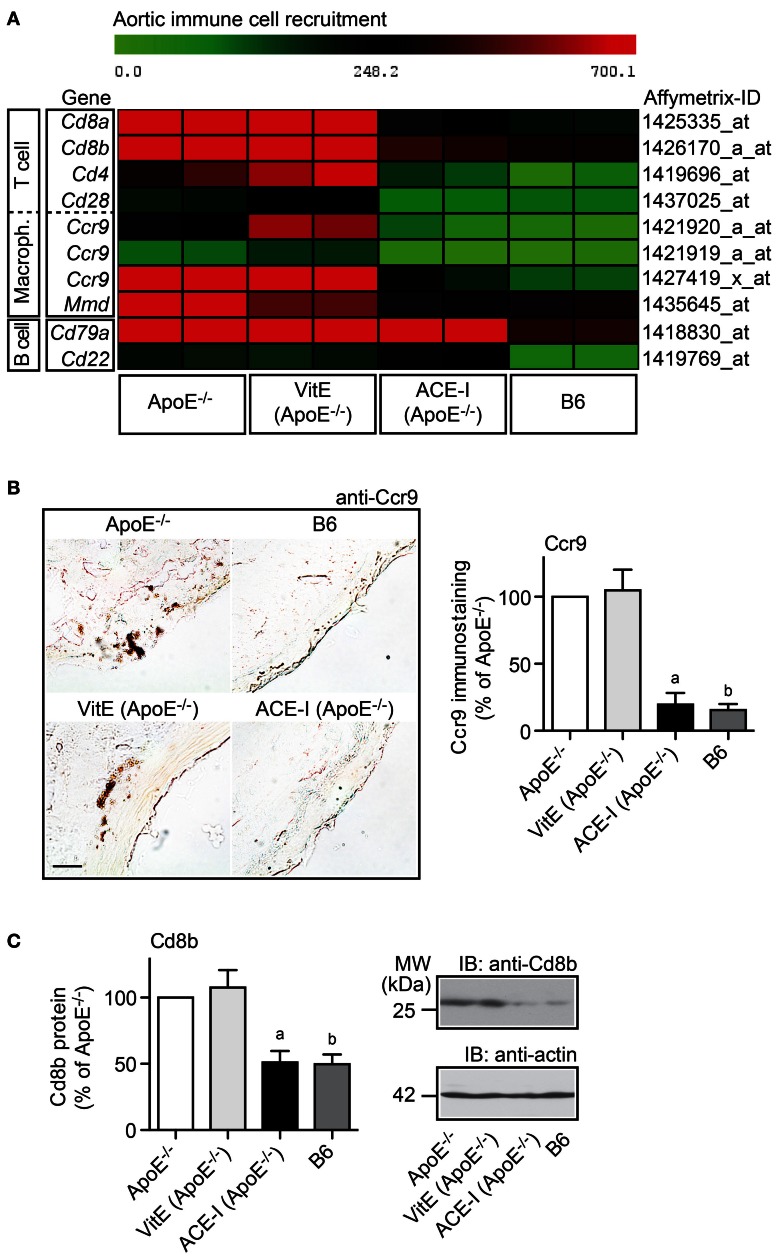
**Pro-inflammatory immune cell recruitment into the atherosclerosis-prone aorta was sensitive to ACE inhibition but insensitive to vitamin E treatment. (A)** Microarray gene expression data of probe sets detecting immune cell-specific markers are presented as heat map centered to the median value. Data filtering and GO analysis identified that genes marking T cells and macrophages were significantly up-regulated in ApoE^−/−^ aortas (≥2-fold; *P* < 0.05) and normalized toward B6 control level by captopril treatment (ACE-I) whereas genes marking B cells, which are considered atheroprotective, were not significantly altered by ACE-inhibitor treatment. In contrast to captopril, vitamin E treatment (VitE) did not reduce the expression of pro-inflammatory immune cell-specific genes in the ApoE^−/−^ aorta. **(B)** Left panels: immunohistological detection of Ccr9-positive aorta-infiltrating immune cells of a 32-week old ApoE^−/−^ mouse and an age-matched vitamin E-treated ApoE^−/−^ mouse confirmed microarray gene expression data. Ccr9-positive cells were not detected in the aorta of a B6 control and an ACE-inhibitor-treated ApoE^−/−^ mouse (bar: 25 μm). The right panel shows quantitative evaluation of immunohistology data from four mice each (±s.d.; *n* = 4; ^a^*P* = 0.0004; ^b^*P* = 0.0001 vs. ApoE^−/−^). **(C)** Left panel: immunoblot quantification of aortic Cd8b protein content of 32-week old ApoE^−/−^ mice, age-matched vitamin E-treated, or ACE-inhibitor-treated ApoE^−/−^ mice, and B6 control mice (±s.d.; *n* = 4; ^a^*P* = 0.0015; ^b^*P* = 0.0009 vs. ApoE^−/−^). The right panels show representative immunoblots.

Angiotensin II AT1 receptor activation exerts a major pro-atherogenic role by stimulating the recruitment of pro-inflammatory immune cells into the atherosclerosis-prone aorta (Abd Alla et al., 2004, 2010; Cassis et al., 2007; Fukuda et al., 2008). To decipher the impact of ACE inhibitor treatment versus vitamin E treatment on immune cell recruitment, microarray data were filtered according to the following criteria: (1) significantly lower gene expression in captopril-treated compared to untreated ApoE^−/−^ mice (*P* ≤ 0.05 and ≤−2-fold down-regulation), (2) membrane localization, and (3) immune cell specificity according to GO analysis. Data filtering identified T cell- and macrophage-specific membrane proteins, which were significantly reduced by captopril treatment toward B6 control level whereas markers of atheroprotective B cells were not decreased (Figure 6A; and Abd Alla et al., 2010). These findings confirm that the recruitment of pro-inflammatory T cells and macrophages into the atherosclerosis-prone aorta of ApoE^−/−^ mice is enhanced by angiotensin II and can be reduced by ACE inhibition (Abd Alla et al., 2010).

In contrast to captopril, antioxidant treatment with vitamin E did not significantly decrease immune-cell-specific markers in the atherosclerosis-prone aorta (Figure 6A). Immunohistology analysis confirmed the microarray data for the pro-atherogenic T cell and macrophage-resident chemokine receptor 9, Ccr9 (Figure 6B). Vitamin E treatment did not prevent the appearance of Ccr9-positive cells in the atherosclerosis-prone aorta of ApoE^−/−^ mice whereas the aorta of captopril-treated mice resembled the B6 control and did not show significant Ccr9-positive cells (Figure 6B).

In agreement with inhibition of the aortic recruitment of pro-inflammatory T cells by captopril, ACE inhibitor treatment with captopril prevented the atherosclerosis-related increase in the aortic content of the T cell-specific Cd8b protein of ApoE^−/−^ mice as determined by immunoblotting (Figure 6C). In contrast, vitamin E treatment did not significantly change the amount of Cd8b protein in the atherosclerosis-prone aorta of ApoE^−/−^ mice compared to untreated ApoE^−/−^ mice (Figure 6C). Together these findings present strong evidence that the recruitment of pro-inflammatory immune cells into the aorta of ApoE^−/−^ mice is enhanced by angiotensin II and sensitive to ACE inhibition but insensitive to vitamin E treatment.

### ACE-inhibition prevented the atherosclerosis-related down-regulation of perivascular nerve-specific genes of ApoE^−/−^ mice

**Figure 7:**
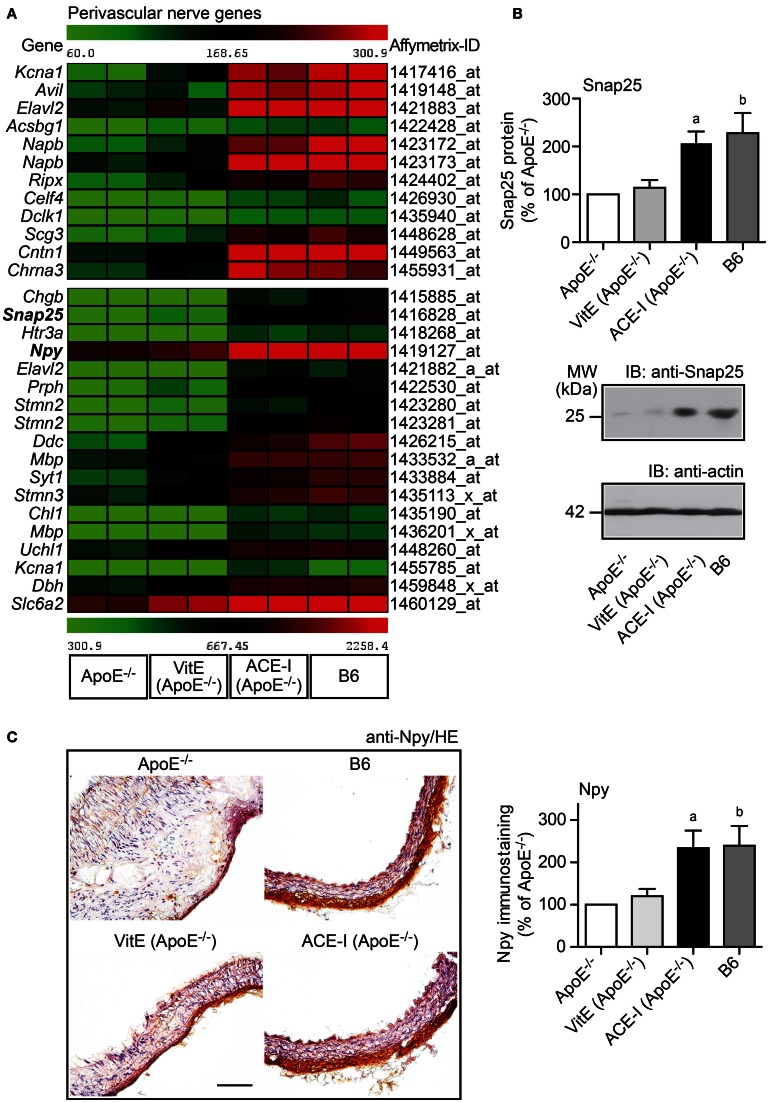
**ACE-inhibition prevented the atherosclerosis-related down-regulation of perivascular nerve-specific genes of ApoE^−/−^ mice. (A)** Microarray gene expression data of probe sets detecting aortic nerve-specific genes, which were significantly down-regulated in ApoE^−/−^ aortas relative B6 controls, are presented as heat map centered to the median value. Data filtering and GO analysis identified 30 significantly down-regulated probe sets in ApoE^−/−^ aortas relative to B6 controls (*P* ≤ 0.01, and ≤-2-fold lower signal intensity) marking the neuronal component (dendrites, myelin sheath, neuronal cell body) and/or the process of nervous system development. Nerve-specific probe sets were not significantly altered by vitamin E treatment (VitE) whereas captopril treatment (ACE-I) normalized the expression of nerve-specific probe sets toward B6 control level. **(B)** Validation of aortic microarray data by quantitative immunoblot analysis of the aortic content of synaptosomal-associated 25 kDa protein, Snap25. The upper panel presents quantitative immunoblot data (±s.d.; *n* = 4; ^a^*P* = 0.0034; ^b^*P* = 0.0090), and middle and lower panels show representative immunoblots. **(C)** Left panels: Immunohistological detection of neuropeptide Y (Npy) showed down-regulation of Npy-positive neurons in the ascending aorta of an ApoE^−/−^ mouse relative to the B6 control (upper panels). Vitamin E treatment (VitE) did not substantially prevent the down-regulation of Npy-positive neurons whereas captopril (ACE-I) treatment prevented the decrease in Npy-positive immunoreactivity in the aorta of an ApoE^−/−^ mouse (bar: 100 μm; nuclei were counterstained with hematoxylin, HE). The right panel shows quantitative evaluation of immunohistology data from four mice each (±s.d.; *n* = 4; ^a^*P* = 0.0075; ^b^*P* = 0.0095 vs. ApoE^−/−^).

Cardiovascular diseases involving atherosclerosis, hypertension or diabetes are reported to cause perivascular nerve deficits, which finally lead to perivascular nerve degeneration (Webster et al., 1991; Scott et al., 1992; Verbeuren et al., 1994; Hobara et al., 2005). In view of those studies, we asked whether hypercholesterolemic ApoE^−/−^ mice also develop perivascular nerve degeneration. To identify significantly altered nerve-specific genes, GO analysis was performed searching for genes associated with a neuron-specific component (e.g., dendrite, myelin sheath, or neuronal cell body) and/or the process of neuronal system development. That approach identified 30 significantly altered neuron/nerve-specific probe sets, which were significantly down-regulated more than 2-fold in the atherosclerotic aorta of untreated ApoE^−/−^ mice relative to B6 controls (Figure 7A). Thus, the aorta of atherosclerosis-prone ApoE^−/−^ mice is characterized by a significant down-regulation of neuron-specific genes, which could reflect the development of perivascular nerve degeneration.

Angiotensin II inhibition is reported to prevent perivascular nerve degeneration in spontaneously hypertensive rats with excessive angiotensin II generation (Hobara et al., 2005). Since hypercholesterolemia also triggers the release of angiotensin II (Daugherty et al., 2004), we asked whether captopril treatment prevented the down-regulation of neuron-specific genes in hypercholesterolemic ApoE^−/−^ mice. Microarray gene expression profiling showed that ACE-inhibition prevented the down regulation of all atherosclerosis-associated nerve-specific genes (Figure 7A). In contrast to ACE inhibition, treatment with vitamin E did not substantially affect those nerve-specific genes (Figure 7A).

Microarray data on the atherosclerosis-related down-regulation of nerve-specific genes were validated by immunoblot detection of the synaptosomal-associated 25 kDa protein (Snap25) as a neuronal synapse-specific protein. Immunoblot detection revealed that the Snap25 protein level was reduced by 56.1% in aortic tissue of untreated ApoE^−/−^ mice compared to B6 control aortas whereas the aortic content of Snap25 protein of captopril-treated ApoE^−/−^ mice was preserved at B6 control level (Figure 7B). In contrast to captopril, vitamin E did not prevent the atherosclerosis-related decrease of the aortic Snap25 protein content (Figure 7B).

Neuropeptide Y (Npy) is present in all sympathetic nerves innervating the cardiovascular system (Zukowska-Grojec et al., 1998). Npy and other sympathetic nerve-associated genes [e.g., dopamine β-hydroxylase (Dbh), dopa decarboxylase (Ddc), and norepinephrine transporter (Slc6a2)] were also among those genes, which showed significant down regulation in the atherosclerotic aorta of ApoE^−/−^ mice, indicating that perivascular nerve degeneration could affect peripheral sympathetic nerves (Figure 7A). To validate microarray data of sympathetic nerve-associated genes, we performed immunohistological detection of Npy. Immunohistology analysis of the neuron-specific Npy confirmed the microarray data and showed that Npy-positive neurons were decreased by 58.1% in the atherosclerotic ApoE^−/−^ aorta compared to the B6 control (Figure 7C). Moreover, captopril treatment maintained the presence of Npy-positive neurons in the aortic adventitia whereas the appearance of Npy-positive neurons was significantly reduced in the vitamin E-treated aorta (Figure 7C). Together these findings strongly suggest that atherosclerotic ApoE^−/−^ mice develop perivascular nerve degeneration, which is sensitive to angiotensin II inhibition but insensitive to vitamin E treatment.

## Discussion

Exaggerated generation of ROS is considered to affect major processes during the pathogenesis of atherosclerosis. However, the full impact of ROS on atherogenesis is not clear because inhibition of ROS generation by genetic or pharmacological tools has only modest effects in animal models or patients with atherosclerosis (Lönn et al., 2012; Schramm et al., 2012). To further investigate the role of ROS in atherogenesis, we used atherosclerosis-prone hypercholesterolemic ApoE^−/−^ mice, and performed treatment with the antioxidant vitamin E. The treatment effect of vitamin E was compared with that of the ACE inhibitor captopril because angiotensin II is an important contributor to ROS in the vasculature and cardiovascular system (Garrido and Griendling, 2009). Moreover, the anti-atherogenic potential of ACE inhibitors is well established in animal models and patients (Hayek et al., 1998; Yusuf et al., 2000; Abd Alla et al., 2004; Wassmann et al., 2004). DHE staining showed that both treatment regimens normalized the increased superoxide generation of atherosclerosis-prone ApoE^−/−^ aortas. Superoxide is the major vascular-damaging ROS triggered by angiotensin II and drives the generation of other ROS such as hydrogen peroxide and peroxynitrite (Zafari et al., 1998; Touyz and Schiffrin, 2001; Guzik and Harrison, 2006; Pacher et al., 2007) Therefore, comparable superoxide reduction indicates overlapping antioxidant effects of vitamin E and the ACE inhibitor, captopril. While we detected comparable antioxidant effects of vitamin E and captopril, ACE inhibition was more effective in slowing the development of atherosclerotic plaques, i.e., vitamin E treatment for 7 months retarded atherosclerotic lesion development by 45.8 ± 11.5% whereas the atherosclerotic plaque area of captopril-treated aortas was reduced by 88.1 ± 7.5%. That observation strongly suggests that ACE inhibition could exert ROS-dependent and ROS-independent anti-atherogenic effects.

To identify atherosclerosis-related pathomechanisms with concordant sensitivity to vitamin E treatment and ACE inhibition, we performed whole genome microarray gene expression profiling of aortic genes. Searching for significantly altered probe sets with concordant regulation between vitamin E and captopril, we found that more than 82% of those concordantly regulated probe sets were normalized toward B6 control level. Since treatment-related normalization toward B6 control level could indicate a potential involvement in atherosclerosis lesion development, we focused on those probe sets with concordant regulation between vitamin E and captopril, which showed normalization toward B6 control level.

GO analysis of probe sets with significantly higher expression after treatment compared to untreated ApoE^−/−^ aortas and immunohistology analysis revealed that vitamin E treatment and ACE inhibition prevented the atherosclerosis-related down-regulation of aortic intima genes of ApoE^−/−^ mice. Identified aortic intima genes such as Sprr3 were previously associated with stratified epithelium, and considered to strengthen the aortic intima against biomechanical stress (Young et al., 2005). In this respect our findings are complementary to recent observations, which indicated that ROS is involved in the degeneration of the aortic intima by enhancing the development of endothelial dysfunction (Guzik and Harrison, 2006). Underlying mechanisms could involve inactivation of the atheroprotective nitric oxide (NO), reduced NO synthesis and/or eNOS uncoupling (Li and Förstermann, 2009). Since angiotensin II-induced activation of NADPH oxidases in endothelial cells is an important contributor to the generation of ROS in the vasculature (Chalupsky and Cai, 2005; Doughan et al., 2008), our microarray study strongly suggests that angiotensin II-stimulated ROS generation could exert a substantial role in deteriorating the endothelial layer, although the precise role of identified genes in atherogenesis remains to be determined.

GO analysis of probe sets with significantly lower expression upon treatment compared to untreated ApoE^−/−^ aortas detected that vitamin E and ACE inhibition prevented the up-regulation of muscle-specific genes in the atherosclerosis-prone aorta of ApoE^−/−^ mice. Immunohistology analysis confirmed this finding and showed that up-regulation of muscle-specific genes such as phospholamban in the aortic media correlated with the proliferation of smooth muscle cells close to atherosclerotic plaques, a process, which marks the transition of the contractile to the synthetic phenotype of vascular smooth muscle cells. Since the initial discovery by Griendling et al. (1994), the involvement of ROS-induced angiotensin II generation in proliferation of smooth muscle cells and vascular hypertrophy is supported by numerous *in vitro* and *in vivo* studies (Garrido and Griendling, 2009). Our findings complement those studies by showing that vitamin E treatment and ACE inhibition mediated similar effects concerning the protection of the aortic intima and media in atherosclerosis-prone ApoE^−/−^ mice. Taken together, our findings are compatible with the concept that ROS aggravates the pathogenesis of atherosclerosis. However, the sole inhibition of angiotensin II-induced and NADPH-dependent generation of ROS does not seem sufficient to prevent the development of atherosclerosis (Schramm et al., 2012). Therefore, ACE inhibition could exert additional anti-atherogenic activities. Such anti-atherogenic effects of angiotensin II inhibition seem to be largely blood pressure-independent because lowering of blood pressure without angiotensin II inhibition, e.g., by hydralazine, did not reduce atherosclerotic lesion area of ApoE^−/−^ mice (Hayek et al., 1999).

In search for additional, vitamin E-independent mechanisms, which could contribute to the anti-atherogenic potential of ACE inhibitors, we focused on the activity of angiotensin II to promote the aortic recruitment of pro-inflammatory immune cells (Abd Alla et al., 2004; Cassis et al., 2007; Fukuda et al., 2008). The microarray study and immune techniques demonstrated and confirmed that angiotensin II inhibition reduced the aortic recruitment of pro-inflammatory immune cells (Abd Alla et al., 2010). While the atherosclerosis-promoting activity of angiotensin II-stimulated aortic immune cell recruitment is firmly established (Abd Alla et al., 2004, 2010; Cassis et al., 2007; Fukuda et al., 2008), our current microarray study revealed that the aortic recruitment of pro-inflammatory cells was apparently insensitive to vitamin E treatment because vitamin E did not prevent the atherosclerosis-related increase of immune-cell specific gene expression in the aorta. Since pro-inflammatory immune cells exert a substantial role in atherogenesis (Ross, 1999; Hansson and Libby, 2006), the latter observation could—at least partially—explain major differences in the anti-atherogenic activity of ACE inhibition relative to vitamin E treatment observed in animal models and patients.

In addition to inflammatory immune cell migration, which is a well-established factor in atherogenesis, whole genome microarray gene expression profiling revealed another, largely unrecognized atherosclerosis-related process in ApoE^−/−^ mice, i.e., the degeneration of perivascular nerves of the aortic adventitia. Notably, the atherosclerosis-prone aorta of ApoE^−/−^ mice was characterized by a significant down-regulation of multiple nerve-specific genes. Down-regulation of neuronal genes could affect sympathetic nerves, as reflected by down-regulation of Ddc, Dbh, the norepinephrine transporter Slc6a2, and the sympathetic nerve-associated Npy. Immunohistology confirmed gene expression data and showed that Npy-positive neurons were significantly decreased in the aortic adventitia of ApoE^−/−^ mice.

Down regulation of perivascular nerve gene expression was largely prevented by ACE inhibition with captopril. In contrast, down-regulation of neuronal genes was not substantially affected by vitamin E treatment. These findings could indicate that perivascular nerve degeneration in atherosclerosis could be promoted by excessive angiotensin II generation. Since perivascular nerve degeneration of spontaneously hypertensive rats was also prevented by angiotensin II inhibition (Hobara et al., 2005), angiotensin II and/or high blood pressure seem to exert a common nerve-degenerating effect in the cardiovascular system. In agreement with that notion, perivascular nerve deficits and/or degeneration were detected in different cardiovascular diseases with exaggerated angiotensin II generation such as hypertension, diabetes, and atherosclerosis (Webster et al., 1991; Scott et al., 1992; Verbeuren et al., 1994; Hobara et al., 2005). At present, the pathophysiological role of perivascular nerve degeneration is not fully understood, and additional research in this area is urgently needed. However, some previous studies indicated that the atherosclerosis-related degeneration of perivascular nerves, notably a local decrease in sympathetic nervous activity, could render the vasculature more susceptible to atherosclerosis by increasing the accumulation of collagen and lipids in the vessel wall (Fronek and Turner, 1980), and reducing the adaptive-trophic influence of sympathetic nerves on vascular structure (Bevan, 1984; Shvalev et al., 1996).

Taken together our study showed that the anti-atherogenic potential of ACE inhibition could be partially attributed to antioxidant vitamin E-like effects in the aortic intima and media. However, major atherosclerosis-related activities of angiotensin II inhibition were not sensitive to vitamin E treatment such as prevention of aortic recruitment of pro-inflammatory immune cells and degeneration of perivascular nerves. Those major differences between ACE inhibition and vitamin E treatment could account for the documented anti-atherogenic activity of ACE inhibitors in patients compared to the weak effect of vitamin E.

### Conflict of interest statement

The authors declare that the research was conducted in the absence of any commercial or financial relationships that could be construed as a potential conflict of interest.

## References

[B1] Abd Alla J. Langer A. Elzahwy S. S. Arman-Kalcek G. Streichert T. Quitterer U. (2010). Angiotensin-converting enzyme inhibition down-regulates the pro-atherogenic chemokine receptor 9 (CCR9)-chemokine ligand 25 (CCL25) axis. J. Biol. Chem. 285, 23496–23505 10.1074/jbc.M110.117481 20504763 PMC2906340

[B2] Abd Alla S. Lother H. Langer A. el Faramawy Y. Quitterer U. (2004). Factor XIIIA transglutaminase crosslinks AT1 receptor dimers of monocytes at the onset of atherosclerosis. Cell 119, 343–354 10.1016/j.cell.2004.10.006 15507206

[B3] Bevan R. D. (1984). Trophic effects of peripheral adrenergic nerves on vascular structure. Hypertension 6, III19–III26 10.1161/01.HYP.6.6_Pt_2.III19 6394491

[B4] Cassis L. A. Rateri D. L. Lu H. Daugherty A. (2007). Bone marrow transplantation reveals that recipient AT1a receptors are required to initiate angiotensin II-induced atherosclerosis and aneurysms. Arterioscler. Thromb. Vasc. Biol. 27, 380–386 10.1161/01.ATV.0000254680.71485.92 17158350

[B5] Chalupsky K. Cai H. (2005). Endothelial dihydrofolate reductase: critical for nitric oxide bioavailability and role in angiotensin II uncoupling of endothelial nitric oxide synthase. Proc. Natl. Acad. Sci. U.S.A. 102, 9056–9061 10.1073/pnas.0409594102 15941833 PMC1157015

[B6] Daugherty A. Rateri D. L. Lu H. Inagami T. Cassis L. A. (2004). Hypercholesterolemia stimulates angiotensin peptide synthesis and contributes to atherosclerosis through the AT1A receptor. Circulation 110, 3849–3857 10.1161/01.CIR.0000150540.54220.C4 15596561

[B7] Denger S. Jahn L. Wende P. Watson L. Gerber S. H. Kübler W. (1999). Expression of monocyte chemoattractant protein-1 cDNA in vascular smooth muscle cells: induction of the synthetic phenotype: a possible clue to SMC differentiation in the process of atherogenesis. Atherosclerosis 144, 15–23 10.1016/S0021-9150(99)00033-7 10381273

[B8] Doughan A. K. Harrison D. G. Dikalov S. I. (2008). Molecular mechanisms of angiotensin II-mediated mitochondrial dysfunction: linking mitochondrial oxidative damage and vascular endothelial dysfunction. Circ. Res. 102, 488–496 10.1161/CIRCRESAHA.107.162800 18096818

[B9] Edwards D. H. Li Y. Ellinsworth D. C. Griffith T. M. (2013). The effect of inorganic arsenic on endothelium-dependent relaxation: role of NADPH oxidase and hydrogen peroxide. Toxicology 306, 50–58 10.1016/j.tox.2013.01.019 23384446 PMC3639371

[B10] Fronek K. Turner J. D. (1980). Combined effect of cholesterol feeding and sympathectomy on the lipid content in rabbit aortas. Atherosclerosis 37, 521–528 10.1016/0021-9150(80)90059-3 7458998

[B11] Fu X. Koller S. Abd Alla J. Quitterer U. (2013). Inhibition of G-protein-coupled receptor kinase 2 (GRK2) triggers the growth-promoting mitogen-activated protein kinase (MAPK) pathway. J. Biol. Chem. 288, 7738–7755 10.1074/jbc.M112.428078 23362259 PMC3597814

[B12] Fukuda D. Sata M. Ishizaka N. Nagai R. (2008). Critical role of bone marrow angiotensin II type 1 receptor in the pathogenesis of atherosclerosis in apolipoprotein E deficient mice. Arterioscler. Thromb. Vasc. Biol. 28, 90–96 10.1161/ATVBAHA.107.152363 17962627

[B13] Garrido A. M. Griendling K. K. (2009). NADPH oxidases and angiotensin II receptor signaling. Mol. Cell. Endocrinol. 302, 148–158 10.1016/j.mce.2008.11.003 19059306 PMC2835147

[B14] Gavrila D. Li W. G. McCormick M. L. Thomas M. Daugherty A. Cassis L. A. (2005). Vitamin E inhibits abdominal aortic aneurysm formation in angiotensin II-infused apolipoprotein E-deficient mice. Arterioscler. Thromb. Vasc. Biol. 25, 1671–1677 10.1161/01.ATV.0000172631.50972.0f 15933246 PMC3974107

[B15] Griendling K. K. Minieri C. A. Ollerenshaw J. D. Alexander R. W. (1994). Angiotensin II stimulates NADH and NADPH oxidase activity in cultured vascular smooth muscle cells. Circ. Res. 74, 1141–1148 10.1161/01.RES.74.6.1141 8187280

[B16] Guzik T. J. Harrison D. G. (2006). Vascular NADPH oxidases as drug targets for novel antioxidant strategies. Drug Discov. Today 11, 524–533 10.1016/j.drudis.2006.04.003 16713904

[B17] Hansson G. K. Libby P. (2006). The immune response in atherosclerosis: a double-edged sword. Nat. Rev. Immunol. 6, 508–519 10.1038/nri1882 16778830

[B18] Hayek T. Attias J. Coleman R. Brodsky S. Smith J. Breslow J. L. (1999). The angiotensin-converting enzyme inhibitor, fosinopril, and the angiotensin II receptor antagonist, losartan, inhibit LDL oxidation and attenuate atherosclerosis independent of lowering blood pressure in apolipoprotein E deficient mice. Cardiovasc. Res. 44, 579–587 10.1016/S0008-6363(99)00239-4 10690290

[B19] Hayek T. Attias J. Smith J. Breslow J. L. Keidar S. (1998). Antiatherosclerotic and antioxidative effects of captopril in apolipoprotein E-deficient mice. J. Cardiovasc. Pharmacol. 31, 540–544 10.1097/00005344-199804000-00011 9554802

[B20] Hayek T. Pavlotzky E. Hamoud S. Coleman R. Keidar S. Aviram M. (2003). Tissue angiotensin-converting-enzyme (ACE) deficiency leads to a reduction in oxidative stress and atherosclerosis: studies in ACE-knockout mice type 2. Arterioscler. Thromb. Vasc. Biol. 23, 2090–2096 10.1161/01.ATV.0000098653.74209.C6 14525797

[B21] Hobara N. Gessei-Tsutsumi N. Goda M. Takayama F. Akiyama S. Kurosaki Y. (2005). Long-term inhibition of angiotensin prevents reduction of periarterial innervation of calcitonin gene-related peptide (CGRP)-containing nerves in spontaneously hypertensive rats. Hypertens. Res. 28, 465–474 10.1291/hypres.28.465 16156511

[B22] Keidar S. (1998). Angiotensin, LDL peroxidation and atherosclerosis. Life Sci. 63, 1–11 10.1016/S0024-3205(98)00014-9 9667759

[B23] Li H. Förstermann U. (2009). Prevention of atherosclerosis by interference with the vascular nitric oxide system. Curr. Pharm. Des. 15, 3133–3145 10.2174/138161209789058002 19754387

[B24] Lönn M. E. Dennis J. M. Stocker R. (2012). Actions of “antioxidants” in the protection against atherosclerosis. Free Radic. Biol. Med. 53, 863–884 10.1016/j.freeradbiomed.2012.05.027 22664312

[B25] Maor I. Hayek T. Coleman R. Aviram M. (1997). Plasma LDL oxidation leads to its aggregation in the atherosclerotic apolipoprotein E-deficient mice. Arterioscler. Thromb. Vasc. Biol. 11, 2995–3005 10.1161/01.ATV.17.11.2995 9409286

[B26] McMurray J. Solomon S. Pieper K. Reed S. Rouleau J. Velazquez E. (2006). The effect of valsartan, captopril, or both on atherosclerotic events after acute myocardial infarction: an analysis of the Valsartan in Acute Myocardial Infarction Trial (VALIANT). J. Am. Coll. Cardiol. 47, 726–733 10.1016/j.jacc.2005.09.055 16487836

[B27] Ozer N. K. Palozza P. Boscoboinik D. Azzi A. (1993). d-alpha-Tocopherol inhibits low density lipoprotein induced proliferation and protein kinase C activity in vascular smooth muscle cells. FEBS Lett. 322, 307–310 10.1016/0014-5793(93)81592-N 8486164

[B28] Pacher P. Beckman J. S. Liaudet L. (2007). Nitric oxide and peroxynitrite in health and disease. Physiol. Rev. 87, 315–424 10.1152/physrev.00029.2006 17237348 PMC2248324

[B29] Piedrahita J. A. Zhang S. H. Hagaman J. R. Oliver P. M. Maeda N. (1992). Generation of mice carrying a mutant apolipoprotein E gene inactivated by gene targeting in embryonic stem cells. Proc. Natl. Acad. Sci. U.S.A. 89, 4471–4475 10.1073/pnas.89.10.4471 1584779 PMC49104

[B30] Plump A. S. Smith J. D. Hayek T. Aalto-Setälä K. Walsh A. Verstuyft J. G. (1992). Severe hypercholesterolemia and atherosclerosis in apolipoprotein E-deficient mice created by homologous recombination in ES cells. Cell 71, 343–353 10.1016/0092-8674(92)90362-G 1423598

[B31] Pratico D. Tangirala R. K. Rader D. J. Rokach J. FitzGerald G. A. (1998). Vitamin E suppresses isoprostane generation *in vivo* and reduces atherosclerosis in ApoE-deficient mice. Nat. Med. 4, 1189–1192 10.1038/2685 9771755

[B32] Ross R. (1999). Atherosclerosis–an inflammatory disease. N. Engl. J. Med. 340, 115–126 10.1056/NEJM199901143400207 9887164

[B33] Schramm A. Matusik P. Osmenda G. Guzik T. J. (2012). Targeting NADPH oxidases in vascular pharmacology. Vascul. Pharmacol. 56, 216–231 10.1016/j.vph.2012.02.012 22405985 PMC3378316

[B34] Scott T. M. Honey A. C. Martin J. F. Booth R. F. (1992). Perivascular innvervation is lost in experimental atherosclerosis. Cardioscience 3, 145–153 1384747

[B35] Shvalev V. N. Kargina-Terent'eva R. A. Kareeva N. I. (1996). The phenomenon of focal sympathetic denervation of the major vessels in the development of atherosclerosis. Morfologiia 110, 102–104 8983494

[B36] Steinberg D. Parthasarathy S. Carew T. E. Khoo J. C. Witztum J. L. (1989). Beyond cholesterol: modifications of low-density lipoprotein that increase its atherogenicity. N. Engl. J. Med. 320, 915–924 10.1056/NEJM198904063201407 2648148

[B37] Suarna C. Hood R. L. Dean R. T. Stocker R. (1993). Comparative antioxidant activity of tocotrienols and other natural lipid-soluble antioxidants in a homogeneous system, and in rat and human lipoproteins. Biochim. Biophys. Acta 1166, 163–170 10.1016/0005-2760(93)90092-N 8443232

[B38] Thomas S. R. Leichtweis S. B. Pettersson K. Croft K. D. Mori T. A. Brown A. J. (2001). Dietary cosupplementation with vitamin E and coenzyme Q(10) inhibits atherosclerosis in apolipoprotein E gene knockout mice. Arterioscler. Thromb. Vasc. Biol. 21, 585–593 10.1161/01.ATV.21.4.585 11304477

[B39] Touyz R. M. Schiffrin E. L. (2001). Increased generation of superoxide by angiotensin II in smooth muscle cells from resistance arteries of hypertensive patients: role of phospholipase D-dependent NAD(P)H oxidase-sensitive pathways. J. Hypertens. 19, 1245–1254 10.1097/00004872-200107000-00009 11446714

[B40] Verbeuren T. J. Simonet S. Herman A. G. (1994). Diet-induced atherosclerosis inhibits release of noradrenaline from sympathetic nerves in rabbit arteries. Eur. J. Pharmacol. 270, 27–34 8157079 10.1016/0926-6917(94)90077-9

[B41] Wassmann S. Czech T. van Eickels M. Fleming I. Böhm M. Nickenig G. (2004). Inhibition of diet-induced atherosclerosis and endothelial dysfunction in apolipoprotein E/angiotensin II type 1A receptor double-knockout mice. Circulation 110, 3062–3067 10.1161/01.CIR.0000137970.47771.AF 15277329

[B42] Webster G. J. Petch E. W. Cowen T. (1991). Streptozotocin-induced diabetes in rats causes neuronal deficits in tyrosine hydroxylase and 5-hydroxytryptamine specific to mesenteric perivascular nerves and without loss of nerve fibers. Exp. Neurol. 113, 53–62 10.1016/0014-4886(91)90146-4 1675174

[B43] Young P. P. Modur V. Teleron A. A. Ladenson J. H. (2005). Enrichment of genes in the aortic intima that are associated with stratified epithelium: implications of underlying biomechanical and barrier properties of the arterial intima. Circulation 111, 2382–2390 10.1161/01.CIR.0000164235.26339.78 15867175

[B44] Yusuf S. Sleight P. Pogue P. Bosch J. Davies R. Dagenais G. (2000). Effects of an angiotensin-converting-enzyme inhibitor, ramipril, on cardiovascular events in high-risk patients. The heart outcomes prevention evaluation study investigators. N. Engl. J. Med. 342, 145–153 10.1056/NEJM200001203420301 10639539

[B45] Zafari A. M. Ushio-Fukai M. Akers M. Yin Q. Shah A. Harrison D. G. (1998). Role of NADH/NADPH oxidase-derived H2O2 in angiotensin II-induced vascular hypertrophy. Hypertension 32, 488–495 10.1161/01.HYP.32.3.488 9740615

[B46] Zhao H. Kalivendi S. Zhang H. Joseph J. Nithipatikom K. Vasquez-Vivar J. (2003). Superoxide reacts with hydroethidine but forms a fluorescent product that is distinctly different from ethidium: potential implications in intracellular fluorescence detection of superoxide. Free Radic. Biol. Med. 34, 1359–1368 10.1016/S0891-5849(03)00142-4 12757846

[B47] Zukowska-Grojec Z. Karwatowska-Prokopczuk E. Rose W. Rone J. Movafagh S. Ji H. (1998). Neuropeptide Y: a novel angiogenic factor from the sympathetic nerves and endothelium. Circ. Res. 83, 187–195 10.1161/01.RES.83.2.187 9686758

